# Abnormal neurite density and orientation dispersion in frontal lobe link to elevated hyperactive/impulsive behaviours in young adults with traumatic brain injury

**DOI:** 10.1093/braincomms/fcac011

**Published:** 2022-01-30

**Authors:** Meng Cao, Yuyang Luo, Ziyan Wu, Kai Wu, Xiaobo Li

**Affiliations:** Department of Biomedical Engineering, New Jersey Institute of Technology, Newark, NJ, USA; Department of Biomedical Engineering, New Jersey Institute of Technology, Newark, NJ, USA; Department of Electrical and Computer Engineering, New Jersey Institute of Technology, Newark, NJ, USA; Department of Electrical and Computer Engineering, School of Biomedical Science and Engineering, South China University of Technology, Guangzhou, China; Department of Biomedical Engineering, New Jersey Institute of Technology, Newark, NJ, USA; Department of Electrical and Computer Engineering, New Jersey Institute of Technology, Newark, NJ, USA

**Keywords:** traumatic brain injury (TBI), attention deficits, neurite orientation dispersion and density imaging (NODDI), neurite density index (NDI), orientation dispersion index (ODI)

## Abstract

Traumatic brain injury is a major public health concern. A significant proportion of individuals experience post-traumatic brain injury behavioural impairments, especially in attention and inhibitory control domains. Traditional diffusion-weighted MRI techniques, such as diffusion tensor imaging, have provided tools to assess white matter structural disruptions reflecting the long-term brain tissue alterations associated with traumatic brain injury. The recently developed neurite orientation dispersion and density imaging is a more advanced diffusion MRI modality, which provides more refined characterization of brain tissue microstructures by assessing the neurite orientation dispersion and neurite density properties. In this study, neurite orientation dispersion and density imaging data from 44 young adults with chronic traumatic brain injury (who had no prior-injury diagnoses of any sub-presentation of attention deficits/hyperactivity disorder or experience of severe inattentive and/or hyperactive behaviours) and 45 group-matched normal controls were investigated, to assess the post-injury morphometrical and microstructural brain alterations and their relationships with the behavioural outcomes. Maps of fractional anisotropy, neurite orientation dispersion index and neurite density index were calculated. Vertex-wise and voxel-wise analyses were conducted for grey matter and white matter, respectively. *Post hoc* region-of-interest-based analyses were also performed. Compared to the controls, the group of traumatic brain injury showed significantly increased orientation dispersion index and significantly decreased neurite density index in various grey matter regions, as well as significantly decreased orientation dispersion index in several white matter regions. Brain–behavioural association analyses indicated that the reduced neurite density index of the left precentral gyrus and the reduced orientation dispersion index of the left superior longitudinal fasciculus were significantly associated with elevated hyperactive/impulsive symptoms in the patients with traumatic brain injury. These findings suggest that post-injury chronical neurite intracellular volume and angular distribution anomalies in the frontal lobe, practically the precentral area, can significantly contribute to the onset of hyperactive/impulsive behaviours in young adults with traumatic brain injury.

## Teaser

Cao *et al.* reported that, relative to controls, young adults with chronic traumatic brain injury (TBI) showed significantly altered neurite density and orientation dispersion in frontal and parietal lobes, and these neural morphometric alterations in the precentral gyrus and superior longitudinal fasciculus play a critical role in post-TBI attention deficits.

## Graphical Abstract

**Graphical Abstract:**
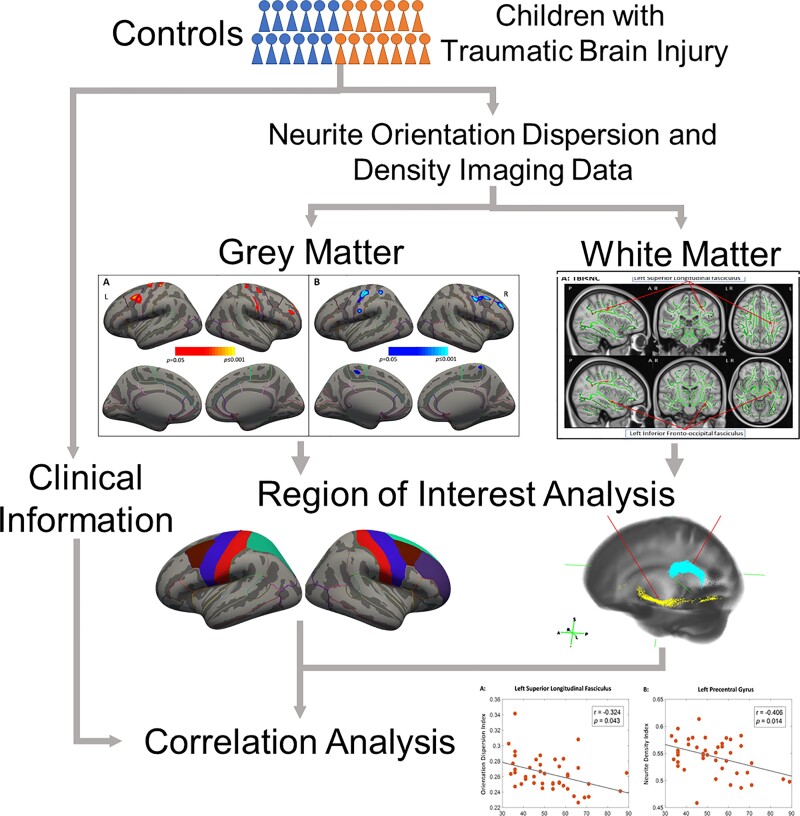

## Introduction

Traumatic brain injury (TBI) is a major public health concern, with ∼2.8 million documented cases occurring each year in the USA,^1^ ∼10% of which are due to sports and recreational activities.^2^ Although the acute symptoms of TBI may be transient and temperate, evidence is emerging that a significant proportion of patients experience long-lasting post-TBI cognitive, emotional, behavioural, sensory and motoric changes.^3^ Among those post-TBI cognitive and behavioural disturbances, attention deficits are the most commonly reported,^4^ with the neuropathological natures remaining unclear.

The long-term post-TBI neural and psychophysiological anomalies have been suggested to relate to traumatic axonal injury that persists in a chronic stage.^5^ The loss of white matter (WM) integrity has been widely considered to play a major role in the clinical phenomenology of TBI.^6^ The pathology of diffuse axonal injury has been widely investigated in diffusion MRI (dMRI)-based approaches, especially diffusion tensor imaging (DTI) that is more sensitive than conventional T_1_- or T_2_-weighted imaging in detecting axonal injury.^7,8^ Among the DTI metrics, fractional anisotropy (FA) has been one of the most commonly reported measures in existing studies in TBI, with findings highly inconsistent in terms of involved brain regions as well as the trends (increase or reduction) of FA abnormalities. In particular, a number of existing studies indicated reduced FA in commonly affected WM tracts, including corticospinal tract,^9^ sagittal stratum^10^ and superior longitudinal fasciculus (SLF)^11^; corona radiata,^12^ uncinated fasciculus,^10,13^ corpus callosum,^12^ inferior longitudinal fasciculus^13^ and cingulum bundle^13^; inferior fronto-occipital fasciculus (IFOF)^14^ among patients with chronic TBI relative to controls. While increased FA in corpus callosum,^15^ internal capsule,^16^ corticospinal tracts,^15^ corona radiate^16^ were also reported in subjects with a history of TBI when compared with matched controls. The discrepancy of these existing findings can partially result from factors associated with the study samples, such as differences in injury severity and mechanism, time since the injury, cognitive and behavioural consequences and sample sizes, as well as techniques implemented for data acquisition and analyses.

The FA value derived from DTI can be influenced by changes in axial diffusivity (AD), radial diffusivity (RD) within the brain tissues or both, making FA sensitive to many types of brain pathology, while incapable of identifying the specific nature of the underlying pathology.^17^ In addition, although DTI can describe water diffusion behaviour at modest *b*-values (e.g. 1000 s/mm^2^), many studies have demonstrated that simple Gaussian diffusion models do not sufficiently describe water diffusion in complex tissues, such as crossing fibres and mixtures of diffusion compartments. Thus, conventional DTI approaches lack the ability to distinguish the spatial organization of neurites. Furthermore, multiple mechanisms, including changes in cell morphology and packing density, or changes in WM fibre orientation, would contribute to the alterations of FA. Recently, a more advanced dMRI modality, the neurite orientation dispersion and density imaging (NODDI), has been developed.^18^ This innovative technique acquires imaging data at multiple levels of diffusion weight, with each sampling many different spatial orientations at high angular resolution. The main parameters derived from NODDI are neurite density index (NDI) and neurite orientation dispersion index (ODI). The intracellular component of NODDI is designed to be representative of both axons and dendrites, thus providing an improved description of grey matter (GM) microstructure. In addition, NODDI enables differentiation of changes to tissue density and tissue ODI (both expressed by FA changes when using DTI), ultimately offering superior sensitivity to micro-level tissue changes. Recently, Churchill *et al.*^19^ conducted a NODDI study in adult athletes with and without TBI and reported significantly increased NDI and decreased ODI in corpus callosum and internal capsule in the TBI group. Besides, another recent study found that compared to conventional DTI, the NODDI parameters were more sensitive imaging biomarkers underlying WM microstructural pathology for relatively short-term (2 weeks and 6 months after TBI) consequences post-mild TBI.^20^ Moreover, significant correlations between NODDI parameters and neurocognitive/behavioural outcomes have also been reported in patients with Alzheimer’s disease,^21^ autism spectrum disorders^22^ and psychosis.^23^ However, the relationships between NODDI measures and TBI-related behaviours alterations have not been sufficiently investigated.

In this study, we proposed to utilize the refined measures provided by NODDI to investigate the neurobiological mechanisms that may underlie TBI-related attention deficits in young adults with chronic (>6 months) TBI. Our previous functional near-infrared spectroscopy (fNIRS) studies showed functional alterations in frontal areas during sustained attention processing in adults with TBI.^24,25^ In addition, our network studies also reported frontal and parietal abnormalities in both functional and structural brain networks in children with TBI.^26,27^ Based on the results of our previous studies, we hypothesized that neural morphometrical and microstructural abnormalities in frontal areas and the major WM fibres connecting frontal and other brain regions may exist, and significantly contribute to post-TBI attention-related behavioural alterations.

## Materials and methods

### Participants

A total of 89 young adults (ranging from 18 to 26 years of age), including 44 patients with TBI (23 males/21 females) and 45 group-matched normal controls (NCs; 23 males/22 females), were initially involved in this study. Participants in the TBI group were recruited from sports teams at New Jersey Institute of Technology (NJIT) and Rutgers University (RU). The NCs were recruited through on-campus flyers at NJIT and RU. The study received Institutional Reviewed Board Approval at both NJIT and RU. Written informed consents were provided by all participants. Within these 89 subjects, 27 were involved in our previous fNIRS study.^24^

The subjects with TBI had a history of one or multiple sports- or recreational activity-related non-penetrating TBIs, with the most recent onset of TBI clinically confirmed at least 6 months prior to the study visit; only had injuries rated as mild to moderate, characterized by the Glasgow Coma Scale (GCS)^28^; had no head injury whichever caused overt focal brain damage; had no history of diagnosis with any sub-presentation of attention-deficit/hyperactivity disorder (ADHD) prior to the first onset TBI. The group of NCs included young adults with no history of head injury; had no history of diagnosis with ADHD and had no severe inattentive and/or hyperactive/impulsive behaviours measured using the Conner’s Adult ADHD Self-Reporting Rating Scales (CAARS, *T*-score <60 for both inattention and hyperactivity/impulsivity subscales) during the study visits. Subjects in both groups were native or fluent speakers of English and strongly right-handed based on the Edinburgh Handedness Inventory. The study excluded subjects who had a history or current diagnosis of any neurological disorders (such as epilepsy); severe psychiatric disorders (including Schizophrenia, Autism Spectrum Disorders, Major Depression, Anxiety, etc.); received treatment with any stimulant or non-stimulant psychotropic medication within the month prior to testing or had contraindications to MRI scanning. Demographic and clinical/behavioural information of the study cohort was included in Table 1.

**Table 1:** Demographic and clinical characteristics in groups of NCs and TBI

	NC (*N* = 40) Mean (SD)	TBI (*N* = 43) Mean (SD)	*P*-value
Age (year)	21.40 (2.31)	21.02 (2.04)	0.432
Education level (year)	14.70 (1.73)	14.23 (1.51)	0.192
Mother’s education level (year)	15.36 (2.21)	15.76 (2.62)	0.467
Father’s education level (year)	15.92 (2.99)	15.81 (2.36)	0.849
Conners’ Adult ADHD Rating Scale (*T*-score)			
Inattentive	45.53 (6.43)	57.67 (15.28)	<0.001
Hyperactive/impulsive	43.25 (5.65)	52.26 (13.72)	<0.001
ADHD total	44.03 (6.14)	56.67 (16.36)	<0.001
Conners’ Adult ADHD Rating Scale (raw score)			
Inattentive	4.50 (2.78)	9.53 (6.40)	<0.001
Hyperactive/impulsive	5.38 (2.59)	9.02 (5.39)	<0.001
ADHD total	9.88 (4.69)	18.44 (10.65)	<0.001
	***N* (%)**	***N* (%)**	***P*-value**
Male	22 (55.0)	23 (53.5)	0.89
Right-handed	40 (100.0)	43 (100.0)	1.00
Race			0.127
Caucasian	13 (32.5)	22 (51.2)	
African American	3 (7.5)	7 (16.3)	
Hispanic/Latino	2 (5.0)	2 (4.7)	
Asian	17 (42.5)	8 (18.6)	
More than one race	5 (12.5)	4 (9.3)	

TBI, group of traumatic brain injury; NC, group of normal controls; SD, standard deviation.

Four subjects were excluded from group-level analysis due to the heavy head motion (with either the mean relative volume-to-volume displacement, maximum rotation or maximum translation >2.5 mm).

### Neuroimaging data acquisition protocol

High-resolution T_1_-weighted structural MRI (0.9 mm^3^ isotropic T_1_w and T_2_w images) and dMRI data were acquired from a 3.0 T Siemens Trio imaging system (Siemens, Erlangen, Germany). The three-dimensional dMRI images were acquired using a three-shell protocol with an echo planar imaging pulse sequence: TR/TE = 7700/103 ms, voxel size = 2.0 mm × 2.0 mm × 2.5 mm, number of slices = 55, FOV = 220 mm × 220 mm × 138 mm. The three shells of dMRI consisted of 64 diffusion-weighting directions at *b* = 300 s/mm^2^, 30 directions at *b* = 700 s/mm^2^ and 10 directions at *b* = 2000 s/mm^2^. In addition, six b0 scans with opposite phase encoding direction were collected for geometric distortion correction. The data of the *b* = 700 s/mm^2^ shell were used for DTI analyses, while data from all three shells were used for NODDI analyses.

### Individual-level imaging data processing

T_1_-weighted MRI data preprocessing were conducted using the Human Connectome Project (HCP) pipeline^29^ and FreeSurfer v6.0.0.^30^ After correction of gradient non-linearity and field intensity, individual white and pial surfaces for surface-based analysis in GM were generated in the native spaces. Then each individual’s pial surface was non-linearly registered to the group-averaged pial surface using multimodal surface matching (MSM) algorithm.^31^

**Figure 1:**
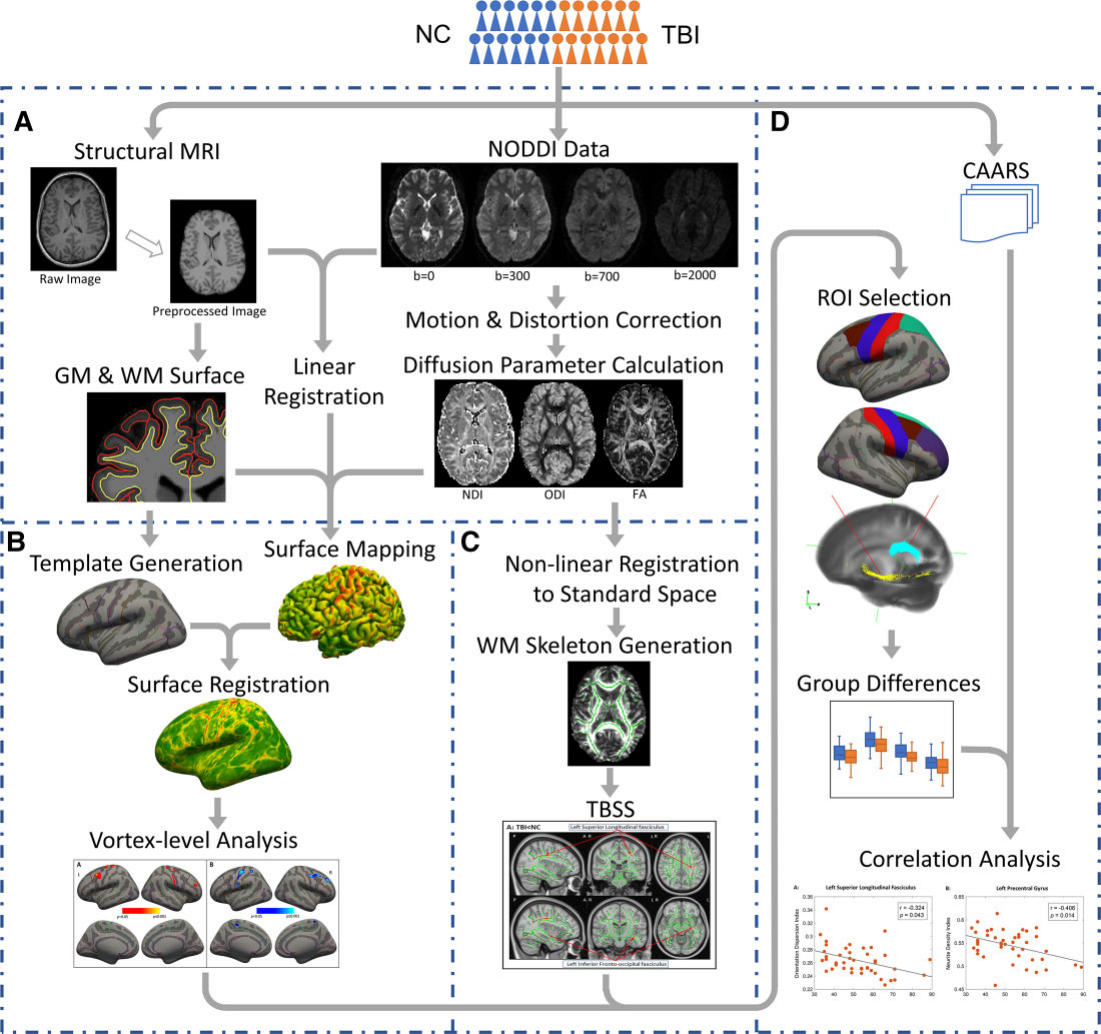
**Overview of the study methods.** (**A**) Individual-level data preprocessing. (**B**) Surface-based analysis of GM. (**C**) Voxel-based analysis of WM. **(D)** Group-level statistical analysis. NC, normal controls; TBI, traumatic brain injury; NODDI, neurite orientation dispersion and density imaging; GM, grey matter; WM, white matter; TBSS, tract-based spatial statistics; CAARS, Conner’s adult attention-deficit/hyperactivity disorder self-reporting rating scales; ROI, region of interest.

The dMRI data preprocessing was performed using Diffusion Toolbox from FMRIB Software Library v6.0 (FSL; www.fmrib.ox.ac.uk/fsl).^32^ Before data preprocessing, the structural MRI and dMRI data from each subject were visually checked for severe head motions and heavy field distortions. Then, the susceptibility-induced field distortions, head motions and eddy current distortion were corrected using *topup* and *eddy* from FSL. Four subjects with heavy head motions were excluded based on the motion parameters estimated by *eddy*. The transformation matrices from local diffusion space to the structural space were calculated by registering a b0 volume to the T_1_-weighted image using Freesurfer’s *BBRegister*.^33^ Voxel-wise NODDI analyses were then conducted using the open-source MATLAB toolbox (http://mig.cs.ucl.ac.uk/index.php?n=Tutorial.NODDImatlab) with the ‘WatsonSHStickTortIsoV_B0’ parameterization to distinguish three microstructural subcomponents: intracellular (neuritic), extracellular and CSF compartments. The NODDI MATLAB toolbox was utilized to generate three-parameter maps: (i) fraction of CSF (*f*_CSF_), which indexes the volume fraction of Gaussian isotropic diffusion (free fluid) within each voxel; (ii) NDI, which indicates the fraction of tissue water restricted within neuritis (axons and dendrites) in the non-CSF compartment. The intracellular signal was modelled as a Watson distribution over cylinders of zero radius, which has a mean orientation vector *μ* and a concentration parameter *κ* ∈ (0, ∞) indicating how much the distribution tends to spread out around *μ* and (iii) ODI, transformed from the concentration parameter, which characterizes spatial configuration of neurites and ranges from 0 (completely parallel neurites) to 1 (completely random neurite orientation). In addition, the *b* = 700 s/mm^2^ data series were analysed to extract voxel-wise conventional DTI measure, FA, by using FSL’s *dtifit*. The individual-level data processing steps are shown in Fig. 1A.

### Surface-based analysis in GM

The surface-based analysis in GM was conducted using the HCP’s connectome workbench and Freesurfer. First, the NDI and ODI maps of each individual were linearly transformed into native structural space. A ribbon mapping method, using WM surface as inner surface and pial surface as the outer surface, was applied to sample the diffusion measures onto each individual’s surface at local space. This method was to construct a polyhedron for each vertex using the vertex’s neighbours, and to calculate the weight of each vertex based on the overlapping volumes with nearby voxels. To generate the vertex-based group difference map, the surface maps of NDI and ODI were subsequently resampled onto the group-averaged surfaces (Fig. 1B) based on MSM surface registration.^31^

### Tract-based spatial statistics analysis in WM

Tract-based spatial statistics (TBSS) of the NDI, ODI and FA maps were carried out in FSL (version 6.0.1). Firstly, FMRIB58_FA in the Montreal Neurological Institute (MNI) common space was used as the target image for non-linear registration of all subjects’ FA maps, using the FMRIB Non-linear Registration Tool (FNIRT; http://fsl.fmrib.ox.ac.uk/fsl/fslwiki/FNIRT/). The transformed FA images in the MNI space were then averaged and skeletonized, to generate the major WM skeleton that would be implemented to all the subjects (Fig. 1C). The threshold of FA ≥ 0.2 was implemented to the average FA map to ensure the WM skeleton to include major WM tracts while exclude peripheral tracts and GM. Each participant’s aligned FA map was then projected onto this skeleton by assigning to each voxel the maximum FA in a line perpendicular to the local skeleton. The NDI, ODI and isotropic compartment were projected onto the mean FA skeleton after applying the warping registration field of each subject to the standard MNI space.

### Statistical analysis

The group comparisons of the vertex-based measures in GM were performed using the Freesurfer’s general linear model (GLM) analysis, with age, sex and parent education level added as covariates. A cluster-wise correction method, with a cluster of 25 mm² or greater and *P* < 0.05, was performed to control for multiple comparisons. The anatomical regions with significant group differences on the GM surface map were then identified using the Desikan–Killiany atlas.^34^

Group comparisons of the voxel-based maps of NDI, ODI and FA in WM were performed in TBSS, using the non-parametric statistical thresholding approach (FSL Randomise permutation algorithm; https://fsl.fmrib.ox.ac.uk/fsl/fslwiki/Randomise). The thresholded mean FA skeleton was used as a mask. Two thousand permutations and statistical inference using threshold-free cluster enhancement (TFCE) were performed, with *P* < 0.05 after family-wise error correction for multiple comparisons. Age, sex and parent education level were used as covariates. The anatomic locations of regions with significant group differences on the WM skeleton were identified from the Johns Hopkins University WM labels atlas.

Based on the results from our previous studies^24–27^ and the result of vertex(voxel)-based analyses, a total of 13 brain regions were selected for a further region-of-interest (ROI)-based analyses. The brain regions consisted of 11 GM ROIs (right rostral middle frontal gyrus, right superior frontal gyrus, left superior parietal lobule, bilateral caudal middle frontal gyrus, bilateral precentral gyrus, bilateral postcentral gyrus and bilateral paracentral lobules) and 2 WM ROIs (left IFOF and left SLF). The GM and WM ROIs were defined based on the Desikan–Killiany atlas and the Johns Hopkins University WM probabilistic tractography atlas, respectively. The mean ODI and NDI values within each ROI were extracted for each subject (no between-group differences in FA were found during the voxel-based analyses). One-way analysis of covariates (ANCOVA) was conducted to identify the between-group differences, with age, sex and parent education level as covariates. Bonferroni’s correction was applied to control multiple comparisons at a significance level of 0.05.

Finally, ROI-based brain imaging measures that showed significant between-group differences were selected for correlation analysis with behavioural measures for clinical symptoms in inattentiveness and hyperactivity/impulsivity. Partial correlation between neuroimaging measures and *T*-scores of the CAARS inattentive and hyperactive/impulsive subscales were conducted in the control and TBI group, respectively. Bonferroni’s correction was applied to control multiple comparisons at a significance level of 0.05.

### Data availability statement

The data that support the findings of this study are available from the corresponding author, upon reasonable request.

## Results

### Demographic, clinical and behavioural measures

The demographic and clinical information of both TBI and NC groups are summarized in Table 1. Demographic measures did not show significant between-group difference. In the TBI group, 23 subjects had one injury, 12 subjects had two injuries, 4 subjects had three injuries and 4 subjects had four injuries. Twenty-four subjects reported loss of consciousness for a short period. Seven subjects reported mild post-traumatic amnesia symptoms. The averaged time between the first injury and the scan was 5.6 (±3.6) years. Compared to the NCs, subjects with TBI showed significantly more clinical symptoms in inattentiveness and hyperactivity/impulsivity measured using the *T*- and raw score of the CAARS inattentive and hyperactive/impulsive subscales. No significant correlations were found between the number of injuries and the CAARS inattentive (*r* = 0.021, *P *= 0.892) or hyperactive/impulsive (*r* = 0.085, *P *= 0.589) subscales.

### Group differences in the intermediate vertex (voxel)-based analyses

**Figure 2:**
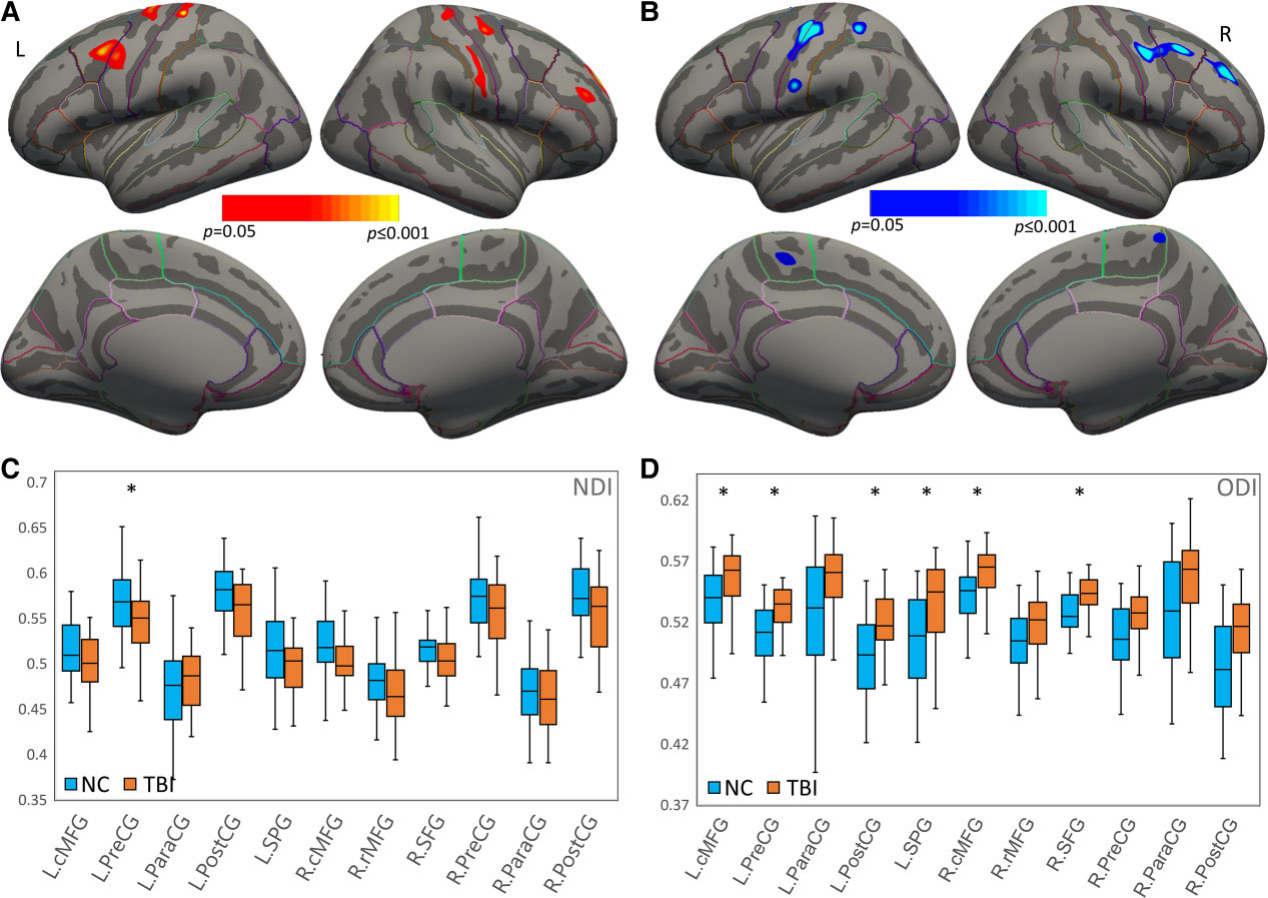
**Surface-based analysis of neurite properties in GM**. (**A**) Surface-based GM analysis showing clusters that have significantly higher NDI in NC than TBI. (**B**) Surface-based GM analysis showing clusters that have significantly lower ODI in NC than TBI. (**C**) ROI-based group comparison in NDI. The TBI group showed significantly decreased NDI in L.PreCG (*F* = 9.903, *P *= 0.026) when compared with controls. (**D**) ROI-based group comparison in ODI. The TBI group showed significantly increased ODI in L.cMFG (*F* = 18.17, *P *< 0.001), L.PreCG (*F* = 19.743, *P *< 0.001), L.PostCG (*F* = 11.296, *P *= 0.014), L.SPG (*F* = 12.813, *P *= 0.007), R.cMFG (*F* = 15.023, *P *= 0.003) and R.SFG (*F* = 16.512, *P *= 0.001) when compared with controls. Regions that showed significant group difference (ANCOVA; *P *< 0.05 after Bonferroni’s correction) were marked with asterisk (*). The bars indicate the maximum and minimum values, the solid horizontal lines in the middle represent the median and the boxes represent the inner quartile range. NDI, neurite density index; ODI, orientation dispersion index; TBI, traumatic brain injury; NC, normal controls; L, left; R, right; cMFG, caudal middle frontal gyrus; PreCG, precentral gyrus; ParaCG, paracentral gyrus; PostCG, postcentral gyrus; SPG, superior parietal gyrus; rMFG, rostral middle frontal gyrus; SFG, superior frontal gyrus.

Group-level results of the surface-based analysis in GM showed significantly decreased NDI in vertex clusters from the bilateral precentral gyrus, bilateral postcentral gyrus, left caudal middle frontal, right superior frontal gyrus and right rostral middle frontal gyrus in patients, relative to controls (Fig. 2A). In addition, the TBI group demonstrated significantly increased ODI in clusters of the bilateral precentral gyrus, bilateral paracentral lobules, left precentral gyrus, left superior parietal lobule and right middle frontal gyrus, when compared with NCs (Fig. 2B). All these regions showed *P* < 0.05 with a cluster >25 mm².

**Figure 3:**
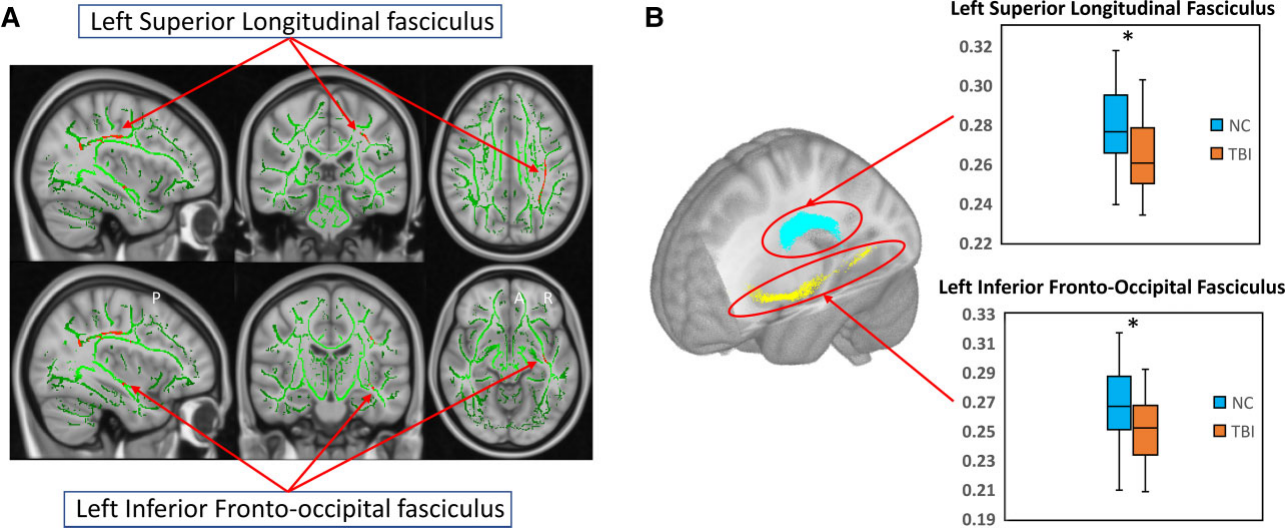
**Voxel-level and ROI analyis of WM**. (**A**) TBSS results showing significant between-group differences of voxel-wise ODI for groups of TBI and NCs. (**B**) ROI analysis results showed significant between-group differences (ANCOVA, *P *< 0.05 after Bonferroni’s correction) in the groups of NC and TBI. The TBI group showed significantly decreased ODI in left SLF (*F* = 6.039, *P *= 0.032) and left IFOF (*F* = 8.115, *P *= 0.012) when compared with controls. *Top* panel presents the results in the left SLF; *bottom* panel presents the results in the left IFOF. The bars indicate the maximum and minimum values, the solid horizontal line in the middle represent the median and the boxes represent the inner quartile range. TBI, traumatic brain injury; NC, normal controls; ODI, orientation dispersion index.

Group-level results of TBSS in WM showed significantly decreased ODI in the left SLF (*P* < 0.05, TFCE corrected) and left IFOF (*P* < 0.05, TFCE corrected) in patients with TBI relative to NCs (Fig. 3A). No significant between-group differences were found in FA or NDI in WM.

### Group differences of ROI-based imaging measures

Compared to controls, the TBI group showed significantly abnormal neurite orientational integrity in bilateral frontal and parietal GM areas, represented by greatly increased ODI in bilateral middle frontal gyri, postcentral, precentral and superior parietal gyri in the left hemisphere, as well as the superior frontal gyrus in the right hemisphere. Relative to controls, the adults with TBI also showed significantly decreased GM neurite density of left precentral gyrus. The ROI-based analyses in WM showed significantly reduced ODI in the left IFOF and SLF in the group of TBI. All the parameters of these results are detailed in Table 2 and graphically depicted in Figs 2C and D and 3B, with all the *P*-values corrected using Bonferroni’s method. Additional ANCOVA was also performed by including race as an additional covariate, and results are included in Supplementary Table 1. No significant effects of race were found on group differences.

**Table 2:** Anatomical regions that showed significant between-group differences of the neurite morphometry

Regions	Measures	NC (*N* = 40) Mean (SD)	TBI (*N* = 43) Mean (SD)	*F*	*P*-value^a^
Grey matter ROIs					
Left caudal middle frontal	ODI	0.537 (0.024)	0.556 (0.022)	18.17	*<*0.001
Left postcentral gyrus	ODI	0.493 (0.035)	0.517 (0.027)	11.296	0.014
Left precentral	ODI	0.509 (0.024)	0.529 (0.022)	19.743	*<*0.001
	NDI	0.568 (0.036)	0.545 (0.033)	9.903	0.026
Left superior parietals	ODI	0.503 (0.039)	0.535 (0.035)	12.813	0.007
Right caudal middle frontal	ODI	0.542 (0.023)	0.559 (0.021)	15.024	0.003
Right superior frontal	ODI	0.529 (0.017)	0.542 (0.015)	16.512	0.001
White matter ROIs					
Left inferior fronto-occipital fasciculus	ODI	0.266 (0.028)	0.251 (0.021)	8.115	0.012
Left superior longitudinal fasciculus	ODI	0.276 (0.022)	0.264 (0.022)	6.039	0.032

ROI, region-of-interest; TBI, group of traumatic brain injury; NC, group of normal controls; SD, standard deviation; ODI, orientation dispersion index; NDI, neurite density index.

^a^ *P*-value after Bonferroni’s correction.

### Brain–behaviour correlations

**Figure 4:**
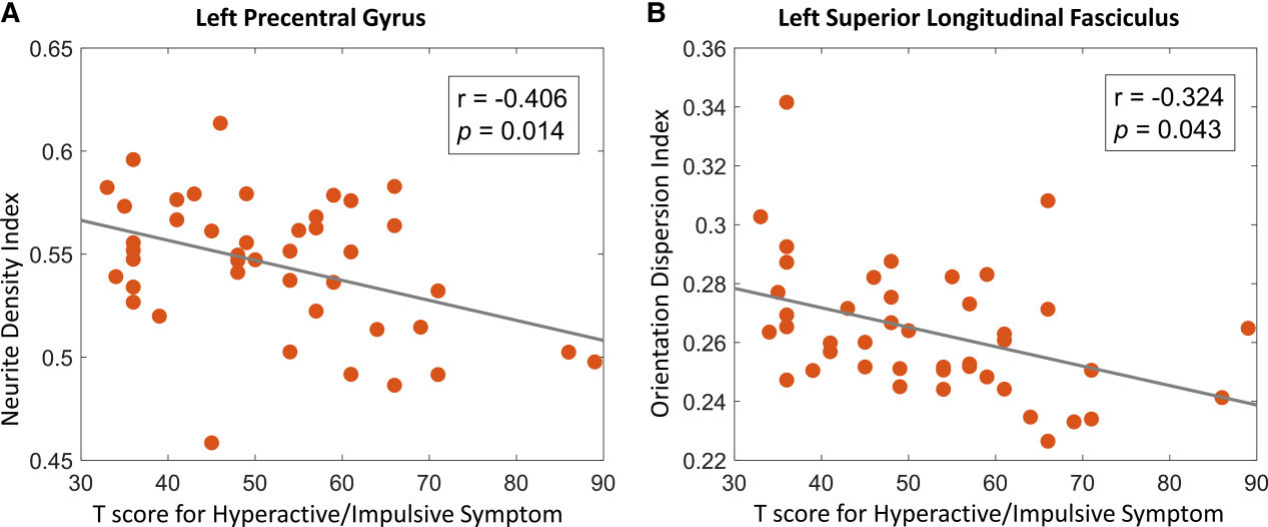
**Brain–behaviour correlation analysis results**. (**A**) Increased NDI of the left precentral gyrus was significantly correlated with reduced hyperactive/impulsive symptom severity *T*-score in the group TBI. (**B**) Greater orientation dispersion index of the left SLF was significantly correlated with reduced hyperactive/impulsive symptom severity *T*-score in the group TBI. TBI, traumatic brain injury.

In the group of TBI, reduced NDI of the left precentral gyrus and reduced ODI of the left SLF were both significantly correlated with elevated hyperactive/impulsive symptoms (Fig. 4). We did not find significant brain–behaviour correlations in the group of NC. Detailed correlation analysis results are included in Supplementary Tables 2 and 3. Brain–behaviour correlations at vortex (voxel)-level are also available in Supplementary Figs 1 and 2.

## Discussion

The present study investigated the neurite morphometry differences, represented by the NDI and ODI in both GM and WM areas, between young adults with TBI and group-matched controls. The results of surface-based GM analysis reported significantly altered neurite morphometry in bilateral frontal and parietal areas, with the left frontal morphometrical abnormalities to be dominated in the subjects with TBI. In the literature of TBI-related studies, structural and functional abnormalities associated with frontal GM areas have been consistently reported in adults and children with TBI. For instance, relative to controls, TBI groups have been found to show significantly decreased frontal cortical thickness^35–37^ and significantly abnormal frontal neural activation during cognitive processing tasks.^38,39^ In addition, cortical GM thinning^40,41^ and functional alterations^38,42^ in the parietal lobe have also been observed in both adults and children with TBI. Along with these existing findings, our results suggest that frontal and parietal GM regions are highly susceptible to the TBI-induced neurite intracellular volume and angular distribution anomalies that can be detected by the advanced NODDI technique, and these neuronal microstructural damages can contribute to the macro-level tissue morphometric and functional abnormalities in subjects with chronic TBI.

Our investigations in WM demonstrated that compared to the matched controls, subjects with TBI had significantly decreased ODI in the left SLF and left IFOF. The SLF and IFOF are two key components of the long-association fronto-parietal pathways, which interconnect cortical and subcortical areas to subserve the visuospatial attention and higher-order cognitive processes.^43,44^ Studies have suggested that SLF and IFOF are critical WM structures involving in the visuospatial orienting and executive components of attention processing,^45^ and brain regions associated with the orienting and executive components of visuospatial attention are most vulnerable to neural damages resulting from mild TBI.^46^ Indeed, substantial previous DTI studies in subjects with TBI have reported structural abnormalities of SLF and IFOF. For instance, significantly increased FA and decreased mean diffusivity (MD) in SLF and/or IFOF have been frequently reported in subjects with a history of sports-related concussion.^15,16,19^ While other studies in subjects with TBI have reported decreased FA and/or increased MD in the left SLF and/or IFOF, and their linkages with prolonged post-TBI behavioural impairments.^47–49^ The inconsistence of these existing findings may be partially explained by the long-term effects of TBI appear to differ from subacute concussion or chronicity of the injury,^50^ the severity level of TBI^51^ and the limitation of DTI measures.^52^

Furthermore, the results of the present study implicated that the significantly decreased NDI of the left precentral gyrus and decreased ODI of the left SLF both significantly contribute to the elevated hyperactive/impulsive symptoms in individuals with TBI. These findings first time in the field suggest that both the TBI-related reduced GM neurite intracellular volume fraction in the left frontal lobe and the overly aligned axons^18^ in the left SLF that anatomically interconnecting frontal and parietal lobes can significantly contribute to TBI-related behavioural impairment in the domain of inhibitory control. The precentral gyrus has been consistently implicated in response inhibition in both primate and human studies.^53–56^ The SLF has also emerged as one of the most reliably identified WM tract underlying inhibitory,^57–60^ and variability in behavioural performances of inhibitory control-related tasks can be partially explained by variability in WM integrity of SLF.^61^ The reduced orientation dispersion in SLF reported in the current study might be caused by decreased branching of major bundles or degeneration of crossing fibres,^15^ which may potentially reduce the communication efficiency in attention-related brain networks during the inhibition process. Together with existing studies, our findings suggest that neurite density and orientation dispersion alterations in the left frontal lobe, especially the precentral area, are significantly vulnerable in TBI-induced chronic neuronal damages; and these neuronal abnormalities significantly link to post-TBI behavioural impairments especially in the domain of inhibitory control.

### Limitations

Although the current study utilized an innovative and robust dMRI technique to study the neurite morphometry and its relationship with post-TBI behavioural impairments in young adults with TBI and have reported significant findings, we acknowledge that there are issues to be addressed.

First, the sex factor associated with post-TBI attention deficits was not investigated due to the sample size limitation of the study. Existing studies indicated that, compared to males, females might have poorer post-TBI behavioural outcomes.^62^ However, our additional analyses in subgroups of females and males with TBI did not show significant differences in the clinical/behavioural and neuroimaging measures. To partially remove the potential effects, we added sex as a fixed effect covariate in the group-level analyses. A future study in a statistically powerful sample is expected to thoroughly investigate the sex-related post-TBI behavioural and anatomical alterations and their interactions. Second, we acknowledge that the diverse injury locations of TBI may be associated with different symptoms. Nevertheless, none of the existing studies have identified any patterns of association between a definitive injury location and long-term deficits in a specific domain of cognitive functions.

## Conclusions

By utilizing an innovative dMRI technique, NODDI, the current study investigated the GM and WM neuronal morphometry and its relationship with post-TBI behavioural impairments in young adults with TBI. The results showed that relative to matched controls, the TBI subjects had significantly altered neurite density and orientation dispersion in frontal and parietal GM areas, and the WM tracts connecting these GM areas. We also found strong relationship of the abnormal left precentral GM NDI and SLF WM ODI with increased hyperactive/impulsive behaviours in the group of TBI. Our findings suggest that significantly altered neurite morphometry exists in frontal and parietal GM regions and the WM major tracts that anatomically connect these GM regions, especially in the left hemisphere; and these neurite disruptions significantly contribute to post-TBI attention problems in subjects with TBI. These findings provide valuable insight into the neuropathological markers of chronic TBI, which have the potential to inform biologically targeted prevention and intervention strategies in affected subjects.

## Supplementary Material

fcac011_Supplementary_Data

## Abbreviations

AD =: axial diffusivity
ADHD =: attention-deficit/hyperactivity disorder
ANCOVA =: one-way analysis of covariates
CAARS =: Conner’s Adult ADHD Self-Reporting Rating Scales
dMRI =: diffusion MRI
DTI =: diffusion tensor imaging
FA =: fractional anisotropy
fNIRS =: functional near-infrared spectroscopy
FNIRT =: FMRIB Non-linear Registration Tool
FSL =: FMRIB Software Library
GCS =: Glasgow Coma Scale
GLM =: general linear model
GM =: grey matter
HCP =: Human Connectome Project
IFOF =: inferior fronto-occipital fasciculus
MD =: mean diffusivity
MNI =: Montreal Neurological Institute
MSM =: multimodal surface matching
NC =: normal control
NDI =: neurite density index
NJIT =: New Jersey Institute of Technology
NODDI =: neurite orientation dispersion and density imaging
ODI =: orientation dispersion index
RD =: radial diffusivity
ROI =: region of interest
RU =: Rutgers University
SLF =: superior longitudinal fasciculus
TBI =: traumatic brain injury
TFCE =: threshold-free cluster enhancement
WM =: white matter

## References

[1] Taylor CA, Bell JM, Breiding MJ, Xu L. Traumatic brain injury-related emergency department visits, hospitalizations, and deaths—United States, 2007 and 2013. MMWR Surveill Summ. 2017;66(9):1–16. 10.15585/mmwr.ss6609a1 PMC5829835 28301451

[2] Centers for Disease Control and Prevention . Nonfatal sports and recreation heat illness treated in hospital emergency departments—United States, 2001–2009. Morb Mortal Wkly Rep 2011;60(29):977–980. 21796094

[3] De Beaumont L, Mongeon D, Tremblay S, et al Persistent motor system abnormalities in formerly concussed athletes. J Athl Train. 2011;46(3):234–240. 21669091 10.4085/1062-6050-46.3.234 PMC3419550

[4] Adeyemo BO, Biederman J, Zafonte R, et al Mild traumatic brain injury and ADHD: A systematic review of the literature and meta-analysis. J Atten Disord. 2014;18(7):576–584. 25047040 10.1177/1087054714543371

[5] Rabinowitz AR, Levin HS. Cognitive sequelae of traumatic brain injury. Psychiatr Clin North Am. 2014;37(1):1–11. 24529420 10.1016/j.psc.2013.11.004 PMC3927143

[6] Hughes DG, Jackson A, Mason DL, Berry E, Hollis S, Yates DW. Abnormalities on magnetic resonance imaging seen acutely following mild traumatic brain injury: Correlation with neuropsychological tests and delayed recovery. Neuroradiology. 2004;46(7):550–558. 15185054 10.1007/s00234-004-1227-x

[7] Hulkower MB, Poliak DB, Rosenbaum SB, Zimmerman ME, Lipton ML. A decade of DTI in traumatic brain injury: 10 years and 100 articles later. Am J Neuroradiol. 2013;34(11):2064–2074. 23306011 10.3174/ajnr.A3395 PMC7964847

[8] Mac Donald CL, Dikranian K, Bayly P, Holtzman D, Brody D. Diffusion tensor imaging reliably detects experimental traumatic axonal injury and indicates approximate time of injury. J Neurosci. 2007;27(44):11869–11876. 17978027 10.1523/JNEUROSCI.3647-07.2007 PMC2562788

[9] Kraus MF, Susmaras T, Caughlin BP, Walker CJ, Sweeney JA, Little DM. White matter integrity and cognition in chronic traumatic brain injury: A diffusion tensor imaging study. Brain. 2007;130(Pt 10):2508–2519. 17872928 10.1093/brain/awm216

[10] Geary EK, Kraus MF, Pliskin NH, Little DM. Verbal learning differences in chronic mild traumatic brain injury. J Int Neuropsychol Soc. 2010;16(3):506–516. 20188015 10.1017/S135561771000010X

[11] Matthews SC, Strigo IA, Simmons AN, O’Connell RM, Reinhardt LE, Moseley SA. A multimodal imaging study in U.S. veterans of Operations Iraqi and Enduring Freedom with and without major depression after blast-related concussion. Neuroimage. 2011;54(Suppl 1):S69–S75. 20451622 10.1016/j.neuroimage.2010.04.269

[12] Churchill NW, Caverzasi E, Graham SJ, Hutchison MG, Schweizer TA. White matter during concussion recovery: Comparing diffusion tensor imaging (DTI) and neurite orientation dispersion and density imaging (NODDI). Hum Brain Mapp. 2019;40(6):1908–1918. 30585674 10.1002/hbm.24500 PMC6865569

[13] Niogi SN, Mukherjee P, Ghajar J, et al Extent of microstructural white matter injury in postconcussive syndrome correlates with impaired cognitive reaction time: A 3 T diffusion tensor imaging study of mild traumatic brain injury. Am J Neuroradiol. 2008;29(5):967–973. 18272556 10.3174/ajnr.A0970 PMC8128563

[14] Singh M, Jeong J, Hwang D, Sungkarat W, Gruen P. Novel diffusion tensor imaging methodology to detect and quantify injured regions and affected brain pathways in traumatic brain injury. Magn Reson Imaging. 2010;28(1):22–40. 19608369 10.1016/j.mri.2009.05.049 PMC2789859

[15] Henry LC, Tremblay J, Tremblay S, et al Acute and chronic changes in diffusivity measures after sports concussion. J Neurotrauma. 2011;28(10):2049–2059. 21864134 10.1089/neu.2011.1836

[16] Sasaki T, Pasternak O, Mayinger M, et al Hockey Concussion Education Project, Part 3. White matter microstructure in ice hockey players with a history of concussion: A diffusion tensor imaging study. J Neurosurg. 2014;120(4):882–890. 24471841 10.3171/2013.12.JNS132092 PMC4863636

[17] Pierpaoli C, Basser PJ. Toward a quantitative assessment of diffusion anisotropy. Magn Reson Med. 1996;36(6):893–906. 8946355 10.1002/mrm.1910360612

[18] Zhang H, Schneider T, Wheeler-Kingshott CA, Alexander DC. NODDI: Practical in vivo neurite orientation dispersion and density imaging of the human brain. Neuroimage. 2012;61(4):1000–1016. 22484410 10.1016/j.neuroimage.2012.03.072

[19] Churchill NW, Caverzasi E, Graham SJ, Hutchison MG, Schweizer TA. White matter microstructure in athletes with a history of concussion: Comparing diffusion tensor imaging (DTI) and neurite orientation dispersion and density imaging (NODDI). Hum Brain Mapp. 2017;38(8):4201–4211. 28556431 10.1002/hbm.23658 PMC6866962

[20] Palacios EM, Owen JP, Yuh EL, et al The evolution of white matter microstructural changes after mild traumatic brain injury: A longitudinal DTI and NODDI study. Sci Adv. 2020;6(32):eaaz6892. 32821816 10.1126/sciadv.aaz6892 PMC7413733

[21] Parker TD, Slattery CF, Zhang J, et al Cortical microstructure in young onset Alzheimer’s disease using neurite orientation dispersion and density imaging. Hum Brain Mapp. 2018;39(7):3005–3017. 29575324 10.1002/hbm.24056 PMC6055830

[22] Andica C, Kamagata K, Kirino E, et al Neurite orientation dispersion and density imaging reveals white matter microstructural alterations in adults with autism. Mol Autism. 2021;12(1):48. 34193257 10.1186/s13229-021-00456-4 PMC8247240

[23] Kraguljac NV, Monroe WS, Anthony T, Jindal RD, Hill H, Lahti AC. Neurite Orientation Dispersion and Density Imaging (NODDI) and duration of untreated psychosis in antipsychotic medication-naïve first episode psychosis patients. Neuroimage Rep. 2021;1(1):100005. 10.1016/j.ynirp.2021.100005 PMC10038586 36969709

[24] Wu Z, Mazzola CA, Catania L, et al Altered cortical activation and connectivity patterns for visual attention processing in young adults post-traumatic brain injury: A functional near infrared spectroscopy study. CNS Neurosci Ther. 2018;24(6):539–548. 29359534 10.1111/cns.12811 PMC6490005

[25] Koenig S, Wu Z, Gao Y, Li X. Abnormal cortical activation in visual attention processing in sub-clinical psychopathic traits and traumatic brain injury: Evidence from an fNIRS study. J Psychopathol Behav Assess. 2020;42(4):627–636.

[26] Cao M, Halperin JM, Li X. Abnormal functional network topology and its dynamics during sustained attention processing significantly implicate post-TBI attention deficits in children. Brain Sci. 2021;11(10):1348. 34679412 10.3390/brainsci11101348 PMC8533973

[27] Cao M, Luo Y, Wu Z, et al Topological aberrance of structural brain network provides quantitative substrates of post-traumatic brain injury attention deficits in children. Brain Connect. 2021;11 (8):651–662. 33765837 10.1089/brain.2020.0866 PMC8817712

[28] Teasdale G, Jennett B. Assessment of coma and impaired consciousness. A practical scale. Lancet. 1974;304(7872):81–84. 10.1016/s0140-6736(74)91639-0 4136544

[29] Glasser MF, Sotiropoulos SN, Wilson JA, et al The minimal preprocessing pipelines for the Human Connectome Project. Neuroimage. 2013;80:105–124. 23668970 10.1016/j.neuroimage.2013.04.127 PMC3720813

[30] Fischl B . FreeSurfer. Neuroimage. 2012;62(2):774–781. 22248573 10.1016/j.neuroimage.2012.01.021 PMC3685476

[31] Robinson EC, Jbabdi S, Glasser MF, et al MSM: A new flexible framework for Multimodal Surface Matching. Neuroimage. 2014;100:414–426. 24939340 10.1016/j.neuroimage.2014.05.069 PMC4190319

[32] Smith SM, Jenkinson M, Woolrich MW, et al Advances in functional and structural MR image analysis and implementation as FSL. Neuroimage. 2004;23(Suppl 1):S208–S219. 15501092 10.1016/j.neuroimage.2004.07.051

[33] Greve DN, Fischl B. Accurate and robust brain image alignment using boundary-based registration. Neuroimage. 2009;48(1):63–72. 19573611 10.1016/j.neuroimage.2009.06.060 PMC2733527

[34] Desikan RS, Segonne F, Fischl B, et al An automated labeling system for subdividing the human cerebral cortex on MRI scans into gyral based regions of interest. Neuroimage. 2006;31(3):968–980. 16530430 10.1016/j.neuroimage.2006.01.021

[35] Mayer AR, Hanlon FM, Ling JM. Gray matter abnormalities in pediatric mild traumatic brain injury. J Neurotrauma. 2015;32(10):723–730. 25313896 10.1089/neu.2014.3534

[36] Turken AU, Herron TJ, Kang X, et al Multimodal surface-based morphometry reveals diffuse cortical atrophy in traumatic brain injury. BMC Med Imaging. 2009;9(20). 10.1186/1471-2342-9-20 PMC2811103 20043859

[37] Wilde EA, Newsome MR, Bigler ED, et al Brain imaging correlates of verbal working memory in children following traumatic brain injury. Int J Psychophysiol. 2011;82(1):86–96. 21565227 10.1016/j.ijpsycho.2011.04.006 PMC3277449

[38] McAllister TW, Sparling MB, Flashman LA, Guerin SJ, Mamourian AC, Saykin AJ. Differential working memory load effects after mild traumatic brain injury. Neuroimage. 2001;14(5):1004–1012. 11697932 10.1006/nimg.2001.0899

[39] Keightley ML, Saluja RS, Chen J-K, et al A functional magnetic resonance imaging study of working memory in youth after sports-related concussion: Is it still working? J Neurotrauma. 2014;31(5):437–451. 24070614 10.1089/neu.2013.3052 PMC3934544

[40] Govindarajan KA, Narayana PA, Hasan KM, et al Cortical thickness in mild traumatic brain injury. J Neurotrauma. 2016;33(20):1809–1817. 26959810 10.1089/neu.2015.4253 PMC5079411

[41] Urban KJ, Riggs L, Wells GD, et al Cortical thickness changes and their relationship to dual-task performance following mild traumatic brain injury in Youth. J Neurotrauma. 2017;34(4):816–823. 27629883 10.1089/neu.2016.4502

[42] Dettwiler A, Murugavel M, Putukian M, Cubon V, Furtado J, Osherson D. Persistent differences in patterns of brain activation after sports-related concussion: A longitudinal functional magnetic resonance imaging study. J Neurotrauma. 2014;31(2):180–188. 23914845 10.1089/neu.2013.2983 PMC3900041

[43] de Schotten MT, Dell’Acqua F, Forkel SJ, et al A lateralized brain network for visuospatial attention. Nat Neurosci. 2011;14(10):1245–1246. 21926985 10.1038/nn.2905

[44] Chechlacz M, Gillebert CR, Vangkilde SA, Petersen A, Humphreys GW. Structural variability within frontoparietal networks and individual differences in attentional functions: An approach using the theory of visual attention. J Neurosci. 2015;35(30):10647–10658. 26224851 10.1523/JNEUROSCI.0210-15.2015 PMC4518045

[45] Fan J, McCandliss BD, Sommer T, Raz A, Posner MI. Testing the efficiency and independence of attentional networks. J Cogn Neurosci. 2002;14(3):340–347. 11970796 10.1162/089892902317361886

[46] Halterman CI, Langan J, Drew A, et al Tracking the recovery of visuospatial attention deficits in mild traumatic brain injury. Brain. 2006;129(Pt 3):747–753. 16330498 10.1093/brain/awh705

[47] Bendlin BB, Ries ML, Lazar M, et al Longitudinal changes in patients with traumatic brain injury assessed with diffusion-tensor and volumetric imaging. Neuroimage. 2008;42(2):503–514. 18556217 10.1016/j.neuroimage.2008.04.254 PMC2613482

[48] Cubon VA, Putukian M, Boyer C, Dettwiler A. A diffusion tensor imaging study on the white matter skeleton in individuals with sports-related concussion. J Neurotrauma. 2011;28(2):189–201. 21083414 10.1089/neu.2010.1430 PMC3037804

[49] Murugavel M, Cubon V, Putukian M, et al A longitudinal diffusion tensor imaging study assessing white matter fiber tracts after sports-related concussion. J Neurotrauma. 2014;31(22):1860–1871. 24786666 10.1089/neu.2014.3368 PMC4224056

[50] Ling JM, Pena A, Yeo RA, et al Biomarkers of increased diffusion anisotropy in semi-acute mild traumatic brain injury: A longitudinal perspective. Brain. 2012;135(Pt 4):1281–1292. 22505633 10.1093/brain/aws073 PMC3326260

[51] Wilde EA, McCauley SR, Hunter JV, et al Diffusion tensor imaging of acute mild traumatic brain injury in adolescents. Neurology. 2008;70(12):948–955. 18347317 10.1212/01.wnl.0000305961.68029.54

[52] Wu Y-C, Mustafi SM, Harezlak J, Kodiweera C, Flashman LA, McAllister TW. Hybrid diffusion imaging in mild traumatic brain injury. J Neurotrauma. 2018;35(20):2377–2390. 29786463 10.1089/neu.2017.5566 PMC6196746

[53] Li C-S, Huang C, Constable RT, Sinha R. Imaging response inhibition in a stop-signal task: Neural correlates independent of signal monitoring and post-response processing. J Neurosci. 2006;26(1):186–192. 16399686 10.1523/JNEUROSCI.3741-05.2006 PMC6674298

[54] Swann N, Tandon N, Canolty R, et al Intracranial EEG reveals a time- and frequency-specific role for the right inferior frontal gyrus and primary motor cortex in stopping initiated responses. J Neurosci. 2009;29(40):12675–12685. 19812342 10.1523/JNEUROSCI.3359-09.2009 PMC2801605

[55] Picazio S, Veniero D, Ponzo V, et al Prefrontal control over motor cortex cycles at beta frequency during movement inhibition. Curr Biol. 2014;24(24):2940–2945. 25484293 10.1016/j.cub.2014.10.043 PMC4274313

[56] Narayanan NS, Laubach M. Top-down control of motor cortex ensembles by dorsomedial prefrontal cortex. Neuron. 2006;52(5):921–931. 17145511 10.1016/j.neuron.2006.10.021 PMC3995137

[57] Cristofori I, Zhong W, Chau A, Solomon J, Krueger F, Grafman J. White and gray matter contributions to executive function recovery after traumatic brain injury. Neurology. 2015;84(14):1394–1401. 25746558 10.1212/WNL.0000000000001446 PMC4395886

[58] Karlsgodt KH, Bato AA, Blair MA, DeRosse P, Szeszko PR, Malhotra AK. White matter microstructure in the executive network associated with aggression in healthy adolescents and young adults. Soc Cogn Affect Neurosci. 2015;10(9):1251–1256. 25691778 10.1093/scan/nsv015 PMC4560949

[59] Mabbott DJ, Noseworthy M, Bouffet E, Laughlin S, Rockel C. White matter growth as a mechanism of cognitive development in children. Neuroimage. 2006;33(3):936–946. 16978884 10.1016/j.neuroimage.2006.07.024

[60] Urger SE, De Bellis MD, Hooper SR, Woolley DP, Chen SD, Provenzale J. The superior longitudinal fasciculus in typically developing children and adolescents: Diffusion tensor imaging and neuropsychological correlates. J Child Neurol. 2015;30(1):9–20. 24556549 10.1177/0883073813520503 PMC4138302

[61] Li P, Tsapanou A, Qolamreza RR, Gazes Y. White matter integrity mediates decline in age-related inhibitory control. Behav Brain Res. 2018;339:249–254. 29126930 10.1016/j.bbr.2017.11.005 PMC5729101

[62] Broshek DK, Kaushik T, Freeman JR, Erlanger D, Webbe F, Barth JT. Sex differences in outcome following sports-related concussion. J Neurosurg. 2005;102(5):856–863. 15926710 10.3171/jns.2005.102.5.0856

