# Supplementary material for: Abnormal neurite density and orientation dispersion in frontal lobe link to elevated hyperactive/impulsive behaviours in young adults with traumatic brain injury
Source: Brain Commun. 2022 Jan 30;4(1):fcac011. doi: 10.1093/braincomms/fcac011 (PMC8853727; doi:10.1093/braincomms/fcac011)
Supplement: fcac011_Supplementary_Data [file fcac011_supplementary_data.docx]

**Supplementary Work 1:**

Additional one-way analysis of covariates (ANCOVA) was performed to identify between group differences by adding race as a covariate, along with age, sex, and parent educate level. Bonferroni correction was applied to control multiple comparisons at significance level of 0.05. Regions that showed significant between-group differences were the same as regions that have been identified using age, sex, and parent educate level as covariates, as shown in **Supplementary Table 1**.

**Supplementary Table 1:** **Anatomical regions that showed significant between-group differences of the neurite morphometry (with age, sex, race, and parent education level as covariates).**

| **Regions** | **Measures** | **NC (N=40)**  **Mean (SD)** | **TBI (N=43)**  **Mean (SD)** | ***F*** | ***p#*** |
| --- | --- | --- | --- | --- | --- |
| **Gray Matter ROIs** |  |  |  |  |  |
| Left Caudal Middle Frontal | ODI | 0.537 (0.024) | 0.556 (0.022) | 14.693 | *0.003* |
| Left Postcentral Gyrus | ODI | 0.493 (0.035) | 0.517 (0.027) | 9.090 | *0.038* |
| Left Precentral | ODI | 0.509 (0.024) | 0.529 (0.022) | 14.041 | *0.004* |
|  | NDI | 0.568 (0.036) | 0.545 (0.033) | 8.538 | *0.049* |
| Left Superior Parietals | ODI | 0.503 (0.039) | 0.535 (0.035) | 14.467 | *0.003* |
| Right Caudal Middle Frontal | ODI | 0.542 (0.023) | 0.559 (0.021) | 14.871 | *0.003* |
| Right Superior Frontal | ODI | 0.529 (0.017) | 0.542 (0.015) | 12.127 | *0.009* |
| **White Matter ROIs** |  |  |  |  |  |
| Left Inferior Fronto-occipital Fasciculus | ODI | 0.266 (0.028) | 0.251 (0.021) | 6.831 | *0.022* |
| Left Superior Longitudinal Fasciculus | ODI | 0.276 (0.022) | 0.264 (0.022) | 5.187 | *0.050* |

ROI: region-of-interest; TBI: group of traumatic brain injury; NC: group of normal controls; SD: standard deviation; ODI: orientation dispersion index; NDI: neurite density index; *p*#: *p* value after Bonferroni correction.

**Supplementary Work 2:**

Partial correlation analyses of NODDI measures and the T scores of inattentive and hyperactive/impulsive symptoms from the CAARS subscales were performed in the TBI control groups, respectively. Age, gender, and parent education level were included as covariates. Results of all the analyses are shown in **Supplementary Tables 2 and 3.**

**Supplementary Table 2:** **Correlations between NODDI measures and inattentive symptoms**

| **Regions** | **Measures** | **Control Group** | | **TBI Group** | |
| --- | --- | --- | --- | --- | --- |
|  |  | **r** | ***p#*** | **r** | ***p#*** |
| **Gray Matter ROIs** |  |  |  |  |  |
| Left Caudal Middle Frontal | ODI | -0.165 | *0.621* | 0.116 | *0.918* |
| Left Postcentral Gyrus | ODI | -0.082 | *1* | 0.205 | *0.374* |
| Left Precentral | ODI | -0.15 | *0.713* | 0.136 | *0.766* |
|  | NDI | 0.008 | *1* | -0.307 | *0.090* |
| Left Superior Parietals | ODI | -0.104 | *1* | 0.067 | *1* |
| Right Caudal Middle Frontal | ODI | -0.232 | *0.298* | 0.177 | *0.512* |
| Right Superior Frontal | ODI | -0.227 | *0.316* | -0.016 | *1* |
| **White Matter ROIs** |  |  |  |  |  |
| Left Inferior Fronto-occipital Fasciculus | ODI | 0.077 | *1* | 0.084 | *1* |
| Left Superior Longitudinal Fasciculus | ODI | -0.217 | *0.358* | -0.168 | *0.571* |

ROI: region-of-interest; TBI: group of traumatic brain injury; NC: group of normal controls; ODI: orientation dispersion index; NDI: neurite density index; *p*#: *p* value after Bonferroni correction.

**Supplementary Table 3:** **Correlations between NODDI measures and hyperactive/impulsive symptoms**

| **Regions** | **Measures** | **Control Group** | | **TBI Group** | |
| --- | --- | --- | --- | --- | --- |
|  |  | **r** | ***p#*** | **r** | ***p#*** |
| **Gray Matter ROIs** |  |  |  |  |  |
| Left Caudal Middle Frontal | ODI | -0.014 | *1* | 0.138 | *0.758* |
| Left Postcentral Gyrus | ODI | -0.038 | *1* | 0.185 | *0.468* |
| Left Precentral | ODI | -0.111 | *0.992* | 0.158 | *0.626* |
|  | NDI | 0.087 | *1* | -0.406 | *0.014** |
| Left Superior Parietals | ODI | 0.018 | *1* | 0.087 | *1* |
| Right Caudal Middle Frontal | ODI | 0.066 | *1* | 0.188 | *0.456* |
| Right Superior Frontal | ODI | 0.032 | *1* | 0.012 | *1* |
| **White Matter ROIs** |  |  |  |  |  |
| Left Inferior Fronto-occipital Fasciculus | ODI | -0.306 | *0.108* | 0.220 | *0.311* |
| Left Superior Longitudinal Fasciculus | ODI | -0.277 | *0.166* | -0.324 | *0.042** |

ROI: region-of-interest; TBI: group of traumatic brain injury; NC: group of normal controls; ODI: orientation dispersion index; NDI: neurite density index; *p*#: *p* value after Bonferroni correction; *: significant *p* value after correction.

**Supplementary Work 3:**

Partial correlation between neurite orientation dispersion and density imaging (NODDI) measures and the T scores of inattentive and hyperactive/impulsive symptoms from the Conner’s Adult ADHD Self-Reporting Rating Scales (CAARS) subscales were performed at the vertex-level (gray matter) and voxel-level (white matter) in the groups of TBI and controls, respectively. Age, gender, and parent education level were included as covariates. The vertex-based analyses were carried out using Freesurfer’s mri_glmfit, while the voxel-based analyses were performed using FSL’s randomise. Cluster correction method was used to control multiple comparisons in the analyses. In the TBI group, significant negative correlation was found between the hyperactive/impulsive symptom score and the neurite density index (NDI) in left precentral gyri, bilateral middle frontal gyrus, and bilateral superior frontal gyrus, as shown in **Supplementary Figure 1**. The TBI group also showed significant negative correlation between the hyperactive/impulsive symptom score and the orientation dispersion index (ODI) in left superior longitudinal fasciculus, as shown in **Supplementary Figure 2**. No significant results were found in the group of controls.

**
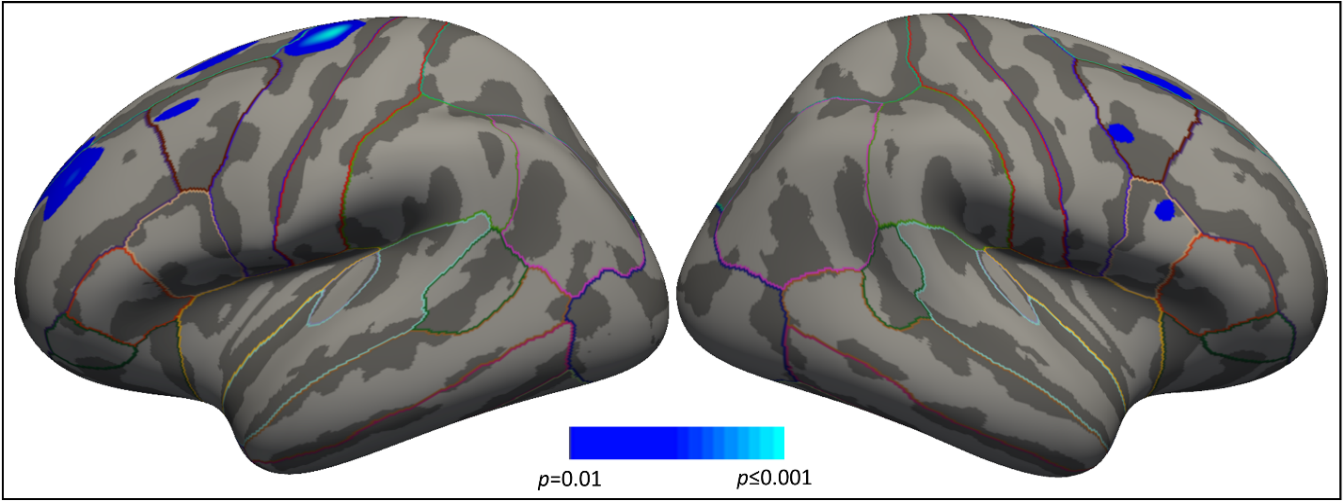
**

**Supplementary Figure 1. Gray matter clusters of the vertex-based NDI that showed significant negative correlation with the hyperactive/impulsive symptom score measured using the CAARS subscale in the group of TBI.**

**
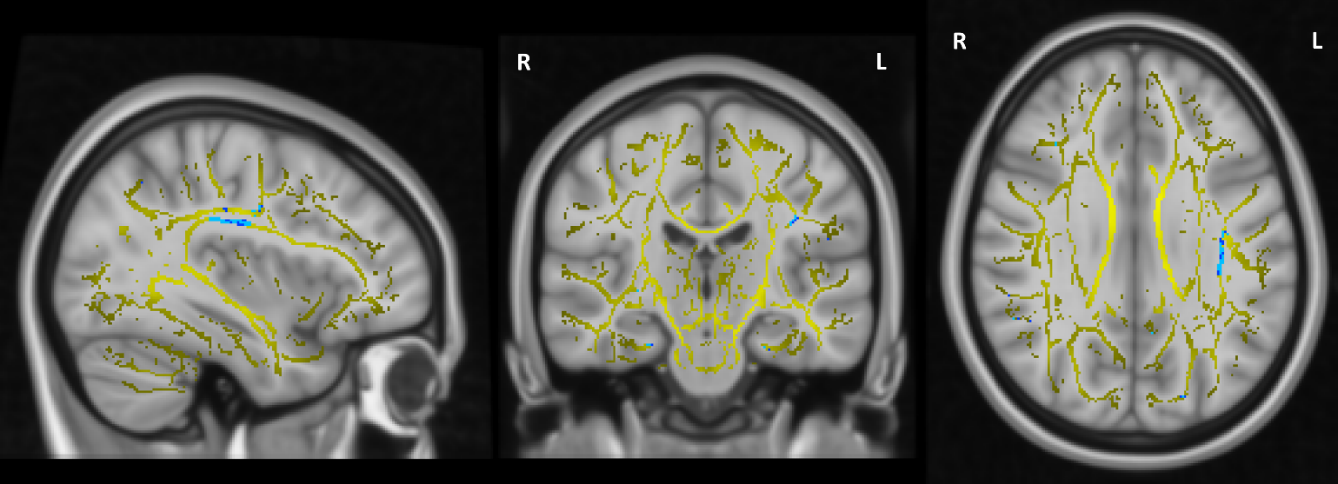
**

**Supplementary Figure 2. White matter clusters of the voxel-based ODI that showed significant negative correlation with the hyperactive/impulsive symptom score measured using the CAARS subscale in the group of TBI (marked in blue with p<0.01).**
